# Toxicities associated with immune checkpoint inhibitors: a systematic study

**DOI:** 10.1097/JS9.0000000000000368

**Published:** 2023-05-03

**Authors:** Xiangyi Kong, Li Chen, Zhaohui Su, Ryan J. Sullivan, Steven M. Blum, Zhihong Qi, Yulu Liu, Yujia Huo, Yi Fang, Lin Zhang, Jidong Gao, Jing Wang

**Affiliations:** a Department of Breast Surgical Oncology, National Cancer Center/National Clinical Research Center for Cancer/Cancer Hospital, Chinese Academy of Medical Sciences and Peking Union Medical College, Beijing, China; b Department of Breast Surgical Oncology, National Cancer Center/National Clinical Research Center for Cancer/Cancer Hospital and Shenzhen Hospital, Chinese Academy of Medical Sciences and Peking Union Medical College, Shenzhen, China; c Department of Thyroid and Breast Surgery, Tongji Hospital, Tongji Medical College of Huazhong University of Science and Technology, Wuhan, Hubei, China; d Suzhou Industrial Park Monash Research Institute of Science and Technology, Suzhou, China; e Clinical Laboratory, Peking Union Medical College Hospital, China; f School of Population Medicine and Public Health, Chinese Academy of Medical Sciences and Peking Union Medical College, Beijing, China; g Fintech Lab, Department of Computer Science, Chow Yei Ching Building, The University of Hong Kong, Pokfulam, Hong Kong Special Administrative Region, China; h Center on Smart and Connected Health Technologies, Mays Cancer Center, School of Nursing, UT Health San Antonio, San Antonio,Texas, United States of America; i Center for Melanoma, Massachusetts General Hospital Cancer Center, Harvard Medical School, Harvard University, Boston, Massachusetts, United States of America; j Department of Medicine-Oncology, Dana-Farber Cancer Institute, Harvard Medical School,Harvard University, Boston, Massachusetts, United States of America; k Melbourne School of Population and Global Health, The University of Melbourne, Melbourne, Victoria, Australia; l The School of Public Health and Preventive Medicine, Monash University, Victoria, Australia

**Keywords:** immune checkpoint inhibitors, immunotherapy, PD-1/PD-L1 inhibitors, randomized controlled trial, systematic review, toxicity

## Abstract

Available evidence shows that the incidence of toxicities associated with cancer immunotherapy, such as programmed cell death 1 (PD-1) and programmed cell death 1 ligand 1 (PD-L1)-related toxicities, is estimated to be between 0.3 and 1.3%.

**Objective:**

This systematic review aimed to investigate cancer patients’ susceptibility to toxicities associated with PD-1/PD-L1 inhibitors and establish a clinically relevant landscape of side effects of PD-1/PD-L1 inhibitors.

**Data sources:**

Relevant publications from PubMed, Embase, Cochrane Library, Web of Science, and China National Knowledge Infrastructure (CNKI) between 2014 and 2019.

**Study eligibility criteria, participants, and interventions:**

We searched randomized controlled trials (RCTs) reporting treatment-related toxicities associated with PD-1 and PD-L1 inhibitors in the treatment of cancers. The primary endpoint was to assess the difference in the incidences of toxicities between cancer patients who did and did not receive PD-1/PD-L1 inhibitors. A total of 29 RCTs, incorporating 8576 patients, met the eligibility criteria.

**Study appraisal and synthesis methods:**

We calculated the pooled relative risks and corresponding 95% CIs using a random-effects model and assessed the heterogeneity between different groups. The subgroup analyses were conducted based on cancer type, toxicity grade (severity), system and organ, treatment regimens in the intervention arm and the control arm, PD-1/PD-L1 inhibitor drug type, and cancer type.

**Results:**

A total of 11 categories (e.g. endocrine toxicity), and 39 toxicity types (e.g. hyperthyroidism) were identified. For toxicities at any grade, those treated with PD-1/PD-L1 inhibitors were at lower risks for gastrointestinal toxicity, hematologic toxicity, and treatment event leading to discontinuation; and were at higher risks for respiratory toxicity (all *P*<0.05). Those treated with PD-1/PD-L1 inhibitors were at lower risks for fatigue, asthenia, and peripheral edema and were at higher risks for pyrexia, cough, dyspnea, pneumonitis, and pruritus.

**Limitations:**

The present research is a meta-analysis at the study level rather than at the patient level; insights on risk factors associated with the development of toxicities cannot be found in our study. There was a possible overlap in Common Terminology Criteria for Adverse Events (CTCAE) definitions which prevents understanding the true rates of specific toxicities.

**Conclusions and implications of key findings:**

For most toxicity types based on system and organ, the incidence proportions for patients in the intervention arm were lower than those in the control arm, which suggested the general safety of PD-1/PD-L1 inhibitors against conventional chemotherapy and cytotoxic t-lymphocyte-associated protein 4 (CTLA-4) inhibitors. Future research should focus on taking effective targeted measures to decrease the risks of different toxicities for different patient populations.

**Systematic review registration number:**

We registered the research protocol with PROSPERO (registration number CRD42019135113).

## Introduction

Highlights

- We systematically investigated toxicities associated with programmed cell death 1 (PD-1)/programmed cell death 1 ligand 1 (PD-L1) inhibitors.
- Twenty-nine randomized controlled trials, incorporating 39 types of toxicities, were analyzed.
- PD-1/PD-L1 inhibitors were at lower risk for gastrointestinal toxicity, etc.
- PD-1/PD-L1 inhibitors were at higher risk for respiratory toxicity, etc.
- PD-1/PD-L1 inhibitors were at lower risk for fatigue, asthenia, etc., and higher risks for pyrexia, cough, etc.

Immunotherapy, especially immune checkpoint inhibitors (PD-1/PD-L1 inhibitors), holds great promise for cancer treatment^1^. Anti-PD-1/PD-L1 strategies have stimulated a paradigm shift in the treatment of oncological malignancies^2,3^. Amongst the promises they hold, PD-1/PD-L1 inhibitors have also fueled the emergence of a new spectrum of toxicities that are different from those associated with traditional chemotherapy agents or monoclonal antibodies^4^, effectively adding new challenges for cancer patients and clinicians to face. Available evidence shows that the incidence of toxicities associated with fatal cancer immunotherapy, such as PD-1/PD-L1-related toxicities, is estimated to be between 0.3 and 1.3%^5^. To reduce or eliminate these toxicities, first, a deep understanding of PD-1/PD-L1 inhibitors is needed.

For starters, PD-1/PD-L1 inhibitors are special in that patients’ susceptibility to their toxicities is not only dependent on the specific agents used in cancer treatments but also on the characteristics of individual patients^4^. Toxicities can affect almost any organ, with varying scales, severities, and frequencies^6^. While these toxicities are usually manageable, they can lead to severe side effects, ranging from treatment withdrawal, fulminant, to fatal events^7^. A thorough understanding of immunotherapy-related toxicities, including the underlying pathogenesis, the kinetics of appearance, and clinical presentation, will help clinicians manage these events more effectively and in turn, improve treatment outcomes^8^. This understanding is particularly needed when factoring in the growing presence of rare toxicities identified in the literature^9–11^, such as neurological toxicities, that can further compound treatment considerations cancer patients, clinicians, and oncologists face.

Since substantial variations exist in different treatment considerations (e.g. cancer histotype, drug and dosing schedule, and toxicity reporting criteria in the publication), ignoring these variations and missing data patterns in toxicity reporting can lead to inaccurate estimation of the actual incidences of treatment-related toxicities associated with PD-1/PD-L1 inhibitors. To shed light on this issue, a growing body of research, including our own, has investigated toxicities associated with PD-1/PD-L1 inhibitors (Supplementary Table S1, Supplemental Digital Content 3, http://links.lww.com/JS9/A415)^12–47^. However, while useful insights are available, substantial gaps can be found in the literature – ranging from incomplete toxicity spectrum presentation of PD-1/PD-L1 inhibitors^48^, to outdated and conflicting findings on patients’ susceptibility to toxicities associated with PD-1/PD-L1 inhibitors^49^. Therefore, to bridge the research gap, in this study, we aim to systematically review and analyze the literature to address the following research objectives: To systematically examine cancer patients’ exposure to toxicities associated with PD-1/PD-L1 inhibitors in light of a series of key treatment considerations mentioned above; To establish a clinically relevant landscape of relative toxicities of PD-1/PD-L1 inhibitors according to the affected system/organ and specific toxicity types.

Overall, the findings of this study can help bridge critical gaps in the literature, as our study is, to date: first, the most comprehensive and systematic examination of cancer patients’ susceptibility to PD-1/PD-L1 inhibitor-associated toxicities – exposure was organized by 9 affected systems (i.e. endocrine toxicity), up to 39 types of toxicities (i.e. hyperthyroidism), and structured across all 4 toxicity grades (i.e. grades 3–4); and second, the most up-to-date and comprehensive analysis of rigorously tested empirical studies (i.e. randomized controlled trials or RCTs) on patients’ susceptibility to toxicities associated with PD-1/PD-L1 inhibitors.

## Meta-analysis process and literature distribution

### Methods

The work has been reported in line with the Preferred Reporting Items for Systematic Reviews and Meta-Analyses (PRISMA, Supplemental Digital Content 1, http://links.lww.com/JS9/A412) and Assessing the methodological quality of systematic reviews (AMSTAR, Supplemental Digital Content 2, http://links.lww.com/JS9/A413) guidelines^50^. We registered the research protocol with PROSPERO (registration number CRD42019135113). Three investigators (X.K., L.C., and L.Z.) independently undertook the literature search, assessment for eligibility, data extraction, and qualitative assessment. Any inconsistencies between the reviewers were resolved by group discussions till a consensus was reached.

### Data sources and searches

A comprehensive literature search was conducted to identify all relevant articles. The databases interrogated were PubMed, Embase, Cochrane Library, Web of Science, and others [China National Knowledge Infrastructure (CNKI)]. The dates searched were from the inception of each database, 31 March 2020. Abstracts and presentations were also reviewed from all major conference proceedings, including the American Society of Clinical Oncology (ASCO) and the European Society for Medical Oncology (ESMO) from January 2010 to March 2020. Two investigators (X.K. and L.C.) independently searched the databases. The search terms included the following keywords: ‘PD-1’, ‘programmed death receptor 1’, ‘PD-L1’, ‘programmed cell death 1 ligand 1’, ‘immunotherapy’, ‘immune checkpoint inhibitor’, ‘durvalumab’, ‘avelumab’, ‘ipilimumab’, ‘tremelimumab’, ‘nivolumab’, ‘pembrolizumab’, and ‘atezolizumab’.

We used the Cochrane highly sensitive search strategies for identifying randomized trials in PubMed and Embase. Search filters used in PubMed (Sensitivity-maximizing version, 2008 revision):

#1 randomized controlled trial [pt]

#2 controlled clinical trial [pt]

#3 randomized [tiab]

#4 placebo [tiab]

#5 drug therapy [sh]

#6 randomly [tiab]

#7 trial [tiab]

#8 groups [tiab]

#9 #1 OR #2 OR #3 OR #4 OR #5 OR #6 OR #7 OR #8

#10 animals [mh] NOT humans [mh]

#11 #9 NOT #10

Search filters used in Embase:

‘crossover procedure’:de OR ‘double-blind procedure’:de OR ‘randomized controlled trial’:de OR ‘single-blind procedure’:de OR (random* OR factorial* OR crossover* OR cross NEXT/1 over* OR placebo* OR doubl* NEAR/1 blind* OR singl* NEAR/1 blind* OR assign* OR allocat* OR volunteer*):de,ab,ti

**Figure 1:**
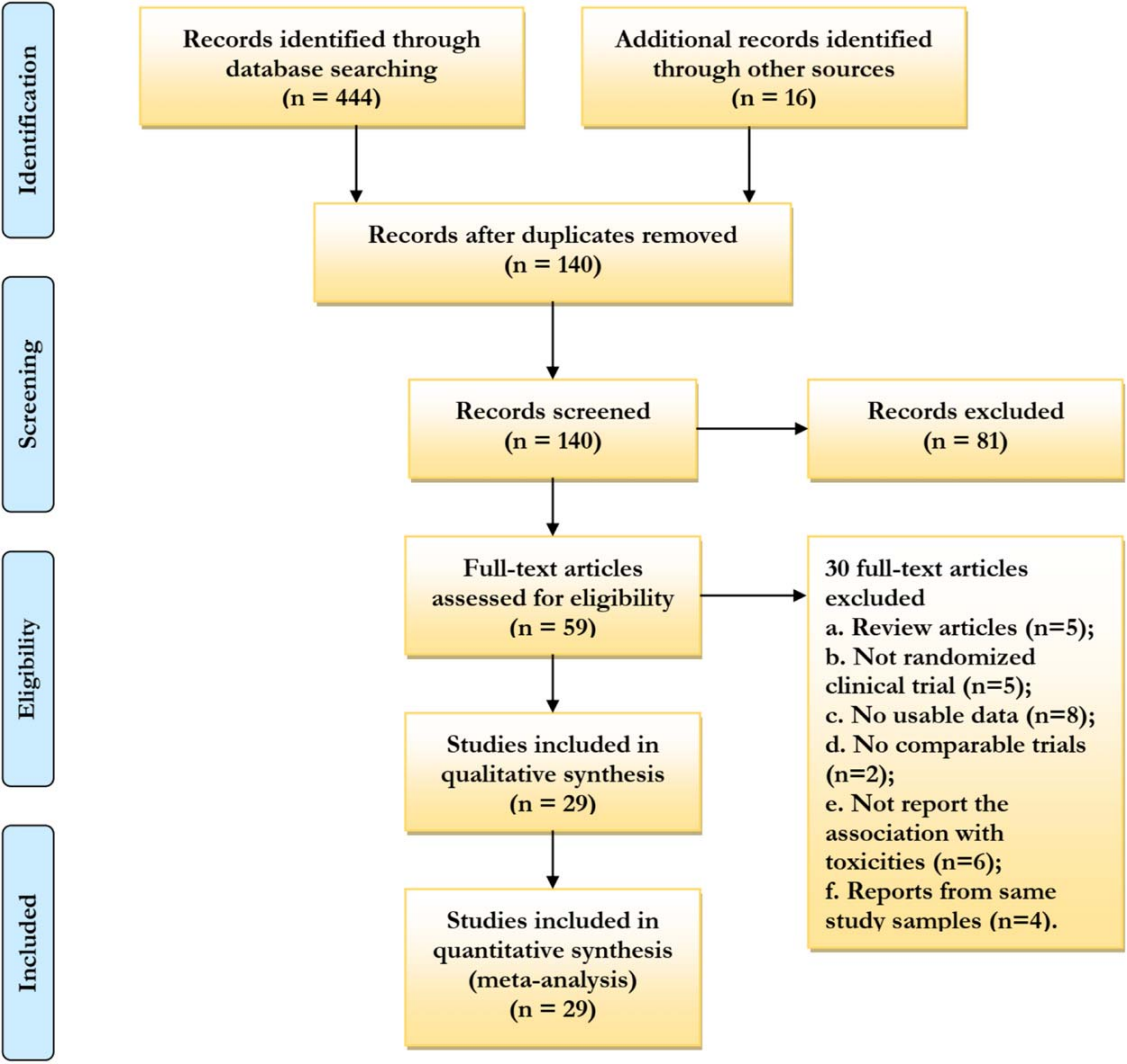
PRISMA (Preferred Reporting Items for Systematic Reviews and Meta-Analyses) flow diagram of study selection for this meta-analysis.

The search was extended by a review of references of articles included in the final selection. We reviewed each publication, and only the most recent and complete report of clinical trials was included. We combined the search results in a bibliographic management tool (EndNote) and used the Bramer method to eliminate duplicates. A literature search and review of reference lists identified 460 relevant publications (Fig. 1).

### Study selection and data extraction

We identified all phase II and III RCTs with PD-1/PD-L1 inhibitors administered alone or in combination with chemotherapy, compared to regimens without PD-1/PD-L1 inhibitors. Details of our inclusion criteria can be found in Supplementary Table S2, Supplemental Digital Content 3, http://links.lww.com/JS9/A415. After screening against eligibility criteria, a total of 29 RCTs (incorporating 8567 patients, published between 2014 and 2019) were included in the final review (Table 1)^51–79^.

**Table 1:** Characteristics of the studies included in this meta-analysis.

									Intervention arm information						Control arm information						
Entry	Author	Year	Country	Institution	Journal	Study phase	Cancer type	Drug name	Patient number	Age	Gender	Any toxicity event	G3–4 toxicity event	Regimen	Patient number	Age	Gender	Any toxicity event	G3–4 toxicity event	Regimen	Reference
1	Robert *et al*.^51^	2014	France	INSERM Unité 981	N Engl J Med	III	Melanoma	Nivolumab	206	64 (18–86)	M 121 vs. F 85	192	70	Nivolumab (at a dose of 3 mg per kilogram of body weight every 2 weeks and dacarbazine-matched placebo every 3 weeks) (206 pts)	205	66 (26–87)	M 125 vs. F 80	194	78	Dacarbazine (at a dose of 1000 mg/m^2^ of the body-surface area every 3 weeks and nivolumab-matched placebo every 2 weeks) (205 pts)	^51^
2	Weber *et al*.^52^	2015	USA	Moffitt Cancer Center	Lancet Oncol	III	Melanoma	Nivolumab	268	59 (23–88)	M 176 vs. F 92	181	24	Nivolumab 3 mg/kg every 2 weeks (268 pts)	102	62 (29–85)	M 85 vs. F 17	81	32	Dacarbazine 1000 mg/m^2^ every 3 weeks or paclitaxel 175 mg/m^2^ combined with carboplatin area under the curve (AUC) 6 every 3 weeks	^52^
3	Larkin *et al*.^53^	2015	USA	Dana-Farber Cancer Institute	N Engl J Med	III	Melanoma	Nivolumab	313	59 (25–90)	M 202 vs. F 111	311	136	Nivolumab (3 mg of nivolumab per kilogram of body weight every 2 weeks (plus ipilimumab matched placebo)) (316 pts)	311	61 (18–89)	M 202 vs. F 109	308	173	Ipilimumab (3 mg of ipilimumab per kilogram every 3 weeks for 4 doses (plus nivolumab-matched placebo) (315 pts)	^53^
4	Borghaei *et al*.^54^	2015	USA	Fox Chase Cancer Center	N Engl J Med	III	Non-small cell lung cancer	Nivolumab	287	61 (37–84)	M 151 vs. F 136	199	30	Nivolumab at a dose of 3 mg per kilogram of body weight every 2 weeks (292 pts)	268	64 (21–85)	M 168 vs. F 100	236	144	Docetaxel at a dose of 75 mg/m^2^ of body-surface area every 3 weeks (290 pts)	^54^
5	Brahmer *et al*.^55^	2015	USA	Sidney Kimmel Comprehensive Cancer Center	N Engl J Med	III	Non-small cell lung cancer	Nivolumab	131	62 (39–85)	M 111 vs. F 20	76	9	Nivolumab at a dose of 3 mg per kilogram of body weight every 2 weeks. (131 pts)	129	64 (42–84)	M 97 vs. F 32	111	77	Docetaxel at a dose of 75 mg/m^2^ of body-surface area every 3 weeks (129 pts)	^55^
6	Ferris *et al*.^56^	2016	USA	Pittsburgh Medical Center Hillman Cancer Center	Oral Oncol	III	Recurrent squamous cell carcinoma	Nivolumab	236	59 (29–83)	M 197 vs. F 39	139	31	Nivolumab (at a dose of 3 mg per kilogram of body weight) every 2 weeks (98 pts)	111	61 (28–78)	M 103 vs. F 8	86	39	Chemotherapy: standard, single-agent systemic therapy (methotrexate, docetaxel, or cetuximab)	^56^
7	Carbone *et al*.^57^	2017	USA	Ohio State University Comprehensive Cancer Center	N Engl J Med	III	Non-small cell lung cancer	Nivolumab	267	63 (32–89)	M 190 vs. F 77	190	47	Nivolumab (administered intravenously at a dose of 3 mg per kilogram of body weight once every 2 weeks) (267 pts)	263	65 (29–87)	M 148 vs. F 115	243	133	Platinum-based chemotherapy (administered once every 3 weeks for up to six cycles (263 pts)	^57^
8	Wolchok *et al*.^58^	2017	USA	Memorial Sloan Kettering Cancer Center and Weill Cornell Medical College	N Engl J Med	III	Advanced melanoma	Nivolumab	313	60 (25–90)	M 202 vs. F 111	270	67	Nivolumab at a dose of 3 mg per kilogram every 2 weeks plus placebo (316 pts)	311	62 (18–89)	M 202 vs. F 109	268	86	Ipilimumab at a dose of 3 mg/kg every 3 weeks for four doses plus placebo, until progression, the occurrence of unacceptable toxic effects, or withdrawal of consent (315 pts)	^58^
9	Hellmann *et al*.^59^	2018	USA	Memorial Sloan Kettering Cancer Center Hospital	N Engl J Med	III	Non-small cell lung cancer	Nivolumab	391	64 (41–87)	M 98 vs. F 293	251	74	Nivolumab (240 mg every 2 weeks) (391 pts)	570	64 (29–80)	M 106 vs. F 464	460	206	Chemotherapy: platinum doublet chemotherapy based on tumor histologic type every 3 weeks for up to four cycles (570 pts)	^59^
10	Hodi *et al*.^60^	2018	USA	Dana-Farber Cancer Institute	Lancet Oncol	III	Advanced melanoma	Nivolumab	313	59 (18–88)	M 206 vs. F 107	270	70	Nivolumab 3 mg/kg every 2 weeks plus placebo (316 pts)	311	61 (18–89)	M 202 vs. F 109	267	86	Ipilimumab 3 mg/kg every 3 weeks for four doses plus placebo (315 pts)	^60^
11	Fradet *et al*.^61^	2019	Canada	CHU de Québec-Université Laval	Ann Oncol	III	Recurrent advanced urothelial cancer	Pembrolizumab	266	67 (29–88)	M 200 vs. F 66	165	44	Pembrolizumab 200 mg every 3 weeks (266 pts)	255	65 (26–84)	M 202 vs. F 53	231	128	Paclitaxel (175 mg/m^2^ i.v. Q3W), docetaxel (75 mg/m^2^ i.v. Q3W), or vinflunine (320 mg/m^2^ i.v. Q3W). (255 pts)	^61^
12	Siu *et al*.^62^	2019	Canada	University of Toronto	JAMA Oncol	II	Head and neck squamous cell carcinoma	Durvalumab	65	62 (23–82)	M 54 vs. F 11	41	8	Durvalumab (10 mg/kg every 2 weeks) (65 pts)	65	61 (42–77)	M 53 vs. F 12	36	11	Tremelimumab (10 mg/kg every 4 weeks for 7 doses, then every 12 weeks for 2 doses)	^62^
13	Martin *et al*.^47^	2019	Germany	Airway Research Center North	J Clin Oncol	III	Advanced non-small-cell lung cancer	Pembrolizumab	154	64.5 (33–90)	M 92 vs. F 62	118	48	Pembrolizumab 200 mg every 3 weeks (for up to 2 years) (154 pts)	150	66.0 (38–85)	M 62 vs. F 88	135	80	Investigator’s choice of platinum-based chemotherapy (four to six cycles) (150 pts)	^63^
14	Barlesi *et al*.^64^	2018	France	Assistance Publique Hôpitaux de Marseille	Lancet Oncol	III	Advanced non-small-cell lung cancer	Avelumab	393	64 (58–69)	M 269 vs. F 124	251	39	Avelumab 10 mg/kg every 2 weeks (393 pts)	365	63 (57–69)	M 273 vs. F 92	313	180	Docetaxel 75 mg/m^2^ every 3 weeks. (365 pts)	^64^
15	Shitara *et al*.^65^	2018	Japan	National Cancer Center Hospital East	Lancet	III	Advanced gastric or gastroesophageal junction cancer	Pembrolizumab	294	62.5 (54–70)	M 202 vs. F 92	155	42	Pembrolizumab 200 mg every 3 weeks for up to 2 years (296 pts)	276	60.0 (53–68)	M 208 vs. F 68	232	96	Standard-dose paclitaxel (276 pts)	^65^
16	Eggermont *et al*.^66^	2018	France	Lyon University	N Engl J Med	III	Resected stage III melanoma	Pembrolizumab	509	54 (19–88)	M 324 vs. F 185	475	161	Pembrolizumab 200 mg every 3 weeks for up to 1 year (509 pts)	502	54 (19–83)	M 304 vs. F 198	453	93	Placebo (502 pts)	^66^
17	Powles *et al*.^67^	2017	UK	Queen Mary University of London	Lancet	III	Locally advanced or metastatic urothelial carcinoma	Atezolizumab	459	67 (33–88)	M 357 vs. F 102	319	0	Atezolizumab 1200 mg every 3 weeks (459 pts)	443	67 (31–84)	M 361 vs. F 82	395	0	Chemotherapy (physician’s choice: vinflunine 320 mg/m^2^, paclitaxel 175 mg/m^2^, or 75 mg/m^2^ docetaxel) intravenously every 3 weeks. (443 pts)	^67^
18	Hamid *et al*.^68^	2016	USA	Angeles Clinic and Research Institute	Eur J Cancer	II	Advanced melanoma	Pembrolizumab	179	60 (27–89)	M 109 vs. F 70	136	30	Pembrolizumab 10 mg/kg intravenously every 3 weeks (179 pts)	171	63 (27–87)	M 114 vs. F 57	138	45	Investigator-choice chemotherapy [carboplatin (eliminated with protocol amendment one), carboplatin plus paclitaxel, dacarbazine, paclitaxel alone, or oral temozolomide] (171 pts)	^68^
19	Herbst *et al*.^69^	2016	USA	Yale Cancer Center and Smilow Cancer Hospital	Lancet	II/III	Advanced non-small cell lung cancer	Pembrolizumab	343	63.0 (56.0–69.0)	M 213 vs. F 130	226	55	Pembrolizumab 10 mg/kg intravenously every 3 weeks (343 pts)	309	62.0 (56.0–69.0)	M 209 vs. F 100	251	109	Docetaxel 75 mg/m^2^ every 3 weeks. (309 pts)	^69^
20	Robert *et al*.^70^	2015	France	Gustave Roussy and Paris-Sud University	N Engl J Med	III	Advanced melanoma	Pembrolizumab	277	63 (22–89)	M 174 vs. F 103	202	28	Pembrolizumab (at a dose of 10 mg per kilogram of body weight) every 3 weeks (277 pts)	256	62 (18–88)	M 162 vs. F 94	187	51	Four doses of Ipilimumab (at 3 mg/kg) every 3 weeks (278 pts)	^70^
21	Weber *et al*.^71^	2017	USA	New York University Perlmutter Cancer Center	N Engl J Med	III	Resected stage III or IV melanoma	Nivolumab	452	56 (19–83)	M 258 vs. F 194	438	115	Nivolumab at a dose of 3 mg per kilogram of body weight every 2 weeks (452 pts)	453	54 (18–86)	M 269 vs. F 184	446	250	Ipilimumab at a dose of 10 mg/kg every 3 weeks for four doses and then every 12 weeks (453 pts)	^71^
22	Ribas *et al*.^72^	2015	USA	University of California	Lancet Oncol	II	Ipilimumab-refractory melanoma	Pembrolizumab	179	60 (27–89)	M 109 vs. F 70	132	25	Pembrolizumab 10 mg/kg intravenously every 3 weeks (179 pts)	171	63 (27–87)	M 109 vs. F 62	138	45	Investigator-choice chemotherapy (paclitaxel plus carboplatin, paclitaxel, carboplatin, dacarbazine, or oral temozolomide). (171 pts)	^72^
23	West *et al*.^74^	2019	USA	Swedish Cancer Institute	Lancet Oncol	III	Non-squamous non-small cell lung cancer	Atezolizumab	473	64 (18–86)	M 277 vs. F 196	471	381	Atezolizumab 1.2 g and carboplatin AUC 6 every 3 weeks (473 pts)	232	65 (38–85)	M 138 vs. F 94	230	164	Carboplatin AUC 6 and nab-paclitaxel every 3 weeks (232 pts)	^73^
24	Langer *et al*.^74^	2016	USA	Abramson Cancer Center of the University of Pennsylvania	Lancet Oncol	II	Advanced, non-squamous non-small cell lung cancer	Pembrolizumab	59	62.5 (54–70)	M 22 vs. F 37	55	23	4 cycles of pembrolizumab 200 mg plus carboplatin AUC 5 mg/ml per min and pemetrexed 500 mg/m^2^ every 3 weeks followed by pembrolizumab for 24 months (59 pts)	62	63.2 (58–70)	M 37 vs. F 25	56	16	Chemotherapy (indefinite pemetrexed maintenance therapy or to 4 cycles of carboplatin and pemetrexed alone followed by indefinite pemetrexed maintenance therapy) intravenously every 3 weeks (62 pts)	^74^
25	Gandhi *et al*.^75^	2018	USA	NYU Perlmutter Cancer Center	N Engl J Med	III	Metastatic non-small-cell lung cancer	Pembrolizumab	405	65.0 (34.0–84.0)	M 254 vs. F 151	404	272	Pemetrexed and a platinum-based drug plus either 200 mg of pembrolizumab every 3 weeks (405 pts)	202	63.5 (34.0–84.0)	M 109 vs. F 93	200	133	Pemetrexed and a platinum-based drug plus placebo every 3 weeks (46 pts)	^75^
26	Socinski *et al*.^76^	2018	Germany	Florida Hospital Cancer Institute	N Engl J Med	III	Metastatic non-squamous non-small cell lung cancer	Atezolizumab	393	63 (31–89)	M 240 vs. F 153	371	230	Atezolizumab plus BCP (ABCP) every 3 weeks for four or six cycles (393 pts)	394	63 (31–90)	M 239 vs. F 155	376	197	Bevacizumab plus carboplatin plus paclitaxel (BCP) every 3 weeks for four or six cycles (394 pts)	^76^
27	Horn *et al*.^77^	2018	USA	Vanderbilt University Medical Center	N Engl J Med	III	Extensive-stage small-cell lung cancer	Atezolizumab	198	64 (28–90)	M 129 vs. F 69	188	115	Carboplatin and etoposide with atezolizumab for four 21-day cycles (198 pts)	196	64 (26–87)	M 132 vs. F 64	181	113	Carboplatin and etoposide with placebo for four 21-day cycles (196 pts)	^77^
28	Paz-Ares *et al*.^78^	2018	Spain	Spanish National Cancer Research Center	N Engl J Med	III	Squamous non-small-cell lung cancer	Pembrolizumab	278	65 (29–87)	M 220 vs. F 58	273	194	200 mg of pembrolizumab on day 1 for up to 35 cycles; all the patients also received carboplatin and either paclitaxel or nanoparticle albumin-bound [nab]-paclitaxel for the first 4 cycles (278 pts)	280	65 (36–88)	M 235 vs. F 45	274	191	Saline placebo for up to 35 cycles; all the patients also received carboplatin and either paclitaxel or nanoparticle albumin-bound [nab]-paclitaxel for the first 4 cycles (280 pts)	^78^
29	Antonia *et al*.^79^	2017	USA	H. Lee Moffitt Cancer Center and Research Institute	N Engl J Med	III	Stage III non-small-cell lung cancer	Durvalumab	475	64 (31–84)	M 334 vs. F 141	460	142	Durvalumab (at a dose of 10 mg per kilogram of body weight intravenously) every 2 weeks for up to 12 months (475 pts)	234	64 (23–90)	M 166 vs. F 68	222	61	Placebo every 2 weeks for up to 12 months (234 pts)	^79^

### Endpoint setting and stratification strategy

Our primary outcome and the parameter compared was the incidence of certain treatment-related toxicities. We used information from the publication and recorded data on toxicities. Where there was ambiguity in terms of data reporting, we directly contacted study authors or pharmaceutical sponsors for additional information. We used the Common Terminology Criteria for Adverse Events (CTCAE) categorization to identify grades 3–4 as severe/life-threatening toxicity and CTCAE grades 1–2 as mild/moderate toxicity. Data from different dosing arms within the same study were extracted and reported separately. A detailed illustration of the stratification strategy we adopted for subgroup analyses can be found in Table 2. Data from all eligible studies were obtained from published manuscripts. The list of cancer histotypes studied were: melanoma (*n*=10), lung cancer (*n*=15), gastrointestinal cancer (*n*=1), genitourinary cancer (*n*=2), and recurrent or metastatic head and neck squamous cell carcinoma (HNSCC) (*n*=1). The PD-1 and PD-L1 inhibitors examined by these RCTs were nivolumab (*n*=11), pembrolizumab (*n*=11), atezolizumab (*n*=4), avelumab (*n*=1), and durvalumab (*n*=2). A detailed literature distribution in terms of various indexes for the included RCTs can be found in Table 3.

**Table 2:** Stratification strategy for subgroup analyses.

Subgroup analysis	Stratification strategy
Subgroup analysis by toxicity grade (severity)	Toxicity grade could be generally divided into two categories, mild/moderate toxicity (grades 1–2) and severe/life-threatening toxicity (grades 3–4). We analyzed all the toxicities together, as well as the grades 3–4 toxicities
Subgroup analysis by affected system/organ	All toxicities together, constitutional toxicity, respiratory toxicity, cutaneous toxicity, gastrointestinal toxicity, hepatotoxicity, hematologic toxicity, neurologic toxicity, endocrine toxicity, inflammatory conditions of cardiac and skeletal muscle (myocarditis and myositis), urinary toxicity, and treatment event leading to discontinuation were analyzed, respectively
Subgroup analysis by treatment regimens in the intervention arm	According to the treatment regimen of the intervention arm, two kinds of randomized control clinical trials were included: (1) single-agent PD-1/PD-L1 inhibitors (22 studies) and (2) PD-1/PD-L1 inhibitors plus chemotherapy (7 studies, all were for lung cancer treatment and the regimens in corresponding control arms were all chemotherapy). In order to avoid the interference effects of the chemotherapy in the intervention arm on the overall results, a subgroup analysis was performed
Subgroup analysis by treatment regimens in the control arm	There were also two kinds of treatment regimens for the control arms: (1) chemotherapy (23 studies) and (2) CTLA-4 inhibitor targeted therapy (6 studies). Considering the possible toxicity differences between conventional chemotherapies and CTLA-4 inhibitors, a subgroup analysis was conducted herein to decrease the cross-confounding and chiasma interference
Subgroup analysis by drug type	Among all the included studies using PD-1 inhibitors, nivolumab, and pembrolizumab were analyzed by subgroup. Because the numbers of included studies that used atezolizumab, durvalumab, or avelumab were small, we analyzed all the PD-L1 inhibitors together rather than separating them
Subgroup analysis by cancer type	Lung cancer and melanoma, with enough included studies, were subgroup-analyzed. Only subgroups, including more than two studies, were considered
Subgroup analysis by follow-up time	We would use the median follow-up time in the intervention arm to indirectly reflect the PD-1/PD-L1 inhibitors exposure time and administered dose to make the corresponding subgroup analysis if the median follow-up time (month) was detected as a factor for the between-study variance in the meta-regression analysis

CTLA-4, cytotoxic t-lymphocyte-associated protein 4; PD-1, programmed cell death 1; PD-L1, programmed cell death 1 ligand 1.

**Table 3:** A detailed literature distribution in terms of various indexes for the included RCTs.

Category items	Data distribution for a total of 29 included studies
Patient numbers	The numbers of patients in the intervention arm and control arm enrolled in each trial ranged between 59 and 509, 62 and 570, respectively
Sex	Of the total 16 173 patients included, 10 311 (63.75%) were male and 5862 (36.25%) were female. Of the total 8576 patients included in the intervention arm, 5491 (64.03%) were male and 3085 (35.97%) were female. Of the total 7597 patients included in the intervention arm, 4820 (63.45%) were male and 2777 (36.55%) were female
Age	The median age of patients ranged from 54 to 67 years old across all included studies
Study phase	24 eligible studies were phase III studies, 5 studies were of phase II
Country	18 studies were conducted in the U.S., which accounted for the largest part, followed by 4 studies conducted in France, 2 studies conducted in Canada, 2 studies conducted in Germany, 1 study conducted in Japan, 1 study conducted in Spain, 1 study conducted in U.K.
Journal	There were 15 studies published in N Engl J Med, which accounted for the most, 6 in Lancet Oncol, and 1 in JAMA Oncol. Other involved journals included Lancet (3 studies), Oral Oncol (1 study), Ann Oncol (1 study), Eur J Cancer (1 study), and J Clin Oncol (1 study)
Intervention arm treatment regimen	22 studies’ intervention arms adopted a treatment regimen of only PD-1/PD-L1 inhibitors, and 7 studies adopted PD-1/PD-L1 inhibitors plus chemotherapy
Control arm treatment regimen	23 studies used regular chemotherapy as the control arm, while 6 studies used CTLA-4 inhibitors
Affected system/organ and toxicity type	
Constitutional toxicity	29 studies reported constitutional toxicity [fatigue (29 studies), asthenia (24 studies), pyrexia (17 studies), headache (9 studies), peripheral edema (10 studies), electrolyte imbalance (1 study)]
Respiratory toxicity	25 studies reported respiratory toxicity [cough (9 studies), dyspnea (14 studies), pneumonitis (25 studies)]
Cutaneous toxicity	26 studies reported cutaneous toxicity [pruritus (23 studies), rash (26 studies), alopecia (14 studies), vitiligo (9 studies), dermatitis (7 studies)]
Gastrointestinal toxicity	29 studies reported gastrointestinal toxicity [nausea (29 studies), vomiting (24 studies), decreased appetite (27 studies), diarrhea (29 studies), constipation (19 studies), colitis (21 studies)]
Hepatotoxicity	16 studies reported hepatotoxicity [increase in alanine aminotransferase (ALT) (16 studies), increase in aspartate aminotransferase (AST) (15 studies), increase in bilirubin (BIU) (7 studies), hepatitis (16 studies)]
Hematologic toxicity	25 studies reported the hematologic toxicity [leukopenia (12 studies), neutropenia (19 studies), thrombocytopenia (10 studies), anemia (25 studies), thrombosis (8 studies)]. 19 studies reported neurotoxic toxicity [myasthenia gravis (2 studies), arthralgia (19 studies), myalgia (13 studies)]
Neurotoxic toxicity	19 studies reported neurotoxic toxicity [myasthenia gravis (2 studies), arthralgia (19 studies), myalgia (13 studies)]
Endocrine toxicity	27 studies reported endocrinal toxicity [hypothyroidism (27 studies), hyperthyroidism (22 studies), adrenal insufficiency (13 studies), hypophysitis (15 studies)]
Inflammatory conditions of cardiac and skeletal muscle	6 studies reported the inflammatory conditions of cardiac and skeletal muscle (myocarditis and myositis)
Urinary toxicity	13 studies reported urinary toxicity (nephritis)
Treatment event leading to discontinuation	28 studies reported the treatment event leading to discontinuation

CTLA-4, cytotoxic t-lymphocyte-associated protein 4; PD-1, programmed cell death 1; PD-L1, programmed cell death 1 ligand 1; RCTs, randomized controlled trials.

### Data synthesis and analysis

We calculated overall event rates by dividing the total number of patients across trials with given toxicity by the total number at risk. We examined the number of events for each immune-related toxicity of interest to determine whether a meta-analysis was feasible. A random-effects model was adopted to calculate the pooled relative risks (RRs) and corresponding 95% CIs to examine statistical and clinical heterogeneity. We used the *I* ^2^ index and the Cochran *Q* statistic to examine heterogeneity across trials for each outcome. If substantial heterogeneity was detected, we performed multivariate meta-regression analyses to explore the proportion of between-study variance. Studies were weighted based on the inverse of the variance of the effect estimate. An estimation of publication bias was evaluated by the Beggs funnel plot. The detailed systematic review process, risk of bias assessment, and quality assessment were shown in Supplementary Table S3, Supplemental Digital Content 3, http://links.lww.com/JS9/A415.

## Incidence proportion of toxicities for PD-1/PD-L1 inhibitors

**Figure 2:**
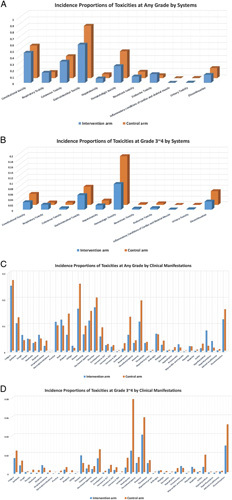
Incidence proportions of toxicities based on the affected systems and toxicity types for both the intervention arm and the control arm. (A) Incidence proportions of toxicities at any grade by systems. (B) Incidence proportions of toxicities at grade 3–4 by systems. (C) Incidence proportions of toxicities at any grade by toxicity types. (D) Incidence proportions of toxicities at grades 3–4 by toxicity types.

The term incidence proportion was defined as the number of toxicities patients were exposed to divided by the total number of patients in the corresponding intervention arm or control arm. As detailed in Supplementary Table S4, Supplemental Digital Content 3, http://links.lww.com/JS9/A415, among the 8576 total patients exposed to PD-1/PD-L1 inhibitors, 6959 (81.15%) had at least one toxicity event of varying degrees and 2510 (29.27%) had at least one toxicity event at grades 3–4. Among the 8576 patients, 5068 (59.10%) of them had gastrointestinal toxicities, followed by constitutional toxicities in 4004 (46.69%) patients and then cutaneous toxicities in 2823 (32.92%) patients. The most common types of grades 3–4 toxicities were hematologic toxicities that had occurred in 807 (9.41%) patients, followed by gastrointestinal toxicity in 458 (5.34%) patients, and then ‘treatment events leading to discontinuation’ in 255 (2.97%) patients. Incidence proportions of toxicities based on the affected systems and toxicity types for both the intervention arm and the control arm were shown in Figure 2A–D. The summary of meta-regression and risk of bias for the included 29 clinical trials with any toxicities were shown in Supplementary Table S5, Supplemental Digital Content 3, http://links.lww.com/JS9/A415 and Supplementary Table S6, Supplemental Digital Content 3, http://links.lww.com/JS9/A415, respectively.

## Toxicity spectrum associated with PD-1/PD-L1 inhibitors

### Overall review of toxicities

A series of analyses were conducted to paint a comprehensive picture of cancer patients’ exposure to toxicities associated with PD-1/PD-L1 inhibitors. Our analyses have taken account of all reported toxicities within a total of 11 categories and of 39 types, as indicated in Supplementary Table S4, Supplemental Digital Content 3, http://links.lww.com/JS9/A415, ranging from fatigue to nephritis. Below, we detailed key findings of our analyses, accompanied by data visualizations that can help readers better understand the results of this study.

For all toxicities of any type, patients treated with PD-1/PD-L1 inhibitors were less likely to experience toxicities when compared with patients treated in control arms (all grades: RR 0.91, 95% CI 0.90–0.93; grades 3–4: RR 0.74, 95% CI 0.71–0.77) (Fig. 3). For toxicities at any grade, those treated with PD-1/PD-L1 inhibitors were at lower risks for gastrointestinal toxicity (RR 0.70, 95% CI 0.63–0.79), hematologic toxicity (RR 0.66, 95% CI 0.51–0.85), and treatment event leading to discontinuation (RR 0.79, 95% CI 0.73–0.85); and were at higher risks for respiratory toxicity (RR 1.79, 95% CI 1.35–2.37). At the specific toxicity type level, those treated with PD-1/PD-L1 inhibitors were at lower risks for fatigue, asthenia, peripheral edema, alopecia, nausea, vomiting, decreased appetite, diarrhea, constipation, colitis, leukopenia, neutropenia, anemia, myalgia, and hypophysitis; and were at higher risks for pyrexia, cough, dyspnea, pneumonitis, pruritus, rash, vitiligo, dermatitis, hepatitis, arthralgia, hypothyroidism, and hyperthyroidism (all *P*<0.05; Supplementary Table S4, Supplemental Digital Content 3, http://links.lww.com/JS9/A415; Fig. 3A). The visualization results for the overall comparison of toxicities of any type at grades 3–4 can be seen in Figure 3B. Those treated with PD-1/PD-L1 inhibitors were at lower risks for constitutional toxicity (grades 3–4), hematologic toxicity (grades 3–4), and treatment events leading to discontinuation (grades 3–4); and were at higher risks for respiratory toxicity (grades 3–4) (all *P*<0.05).

Figure 3Forest plots for the overall comparison of toxicities. (A) Summary relative risks for toxicities at any grade. (B) Summary relative risks for toxicities at grades 3–4. ALT, alanine aminotransferase; AST, aspartate aminotransferase; BIU, bilirubin.

**Figure:**
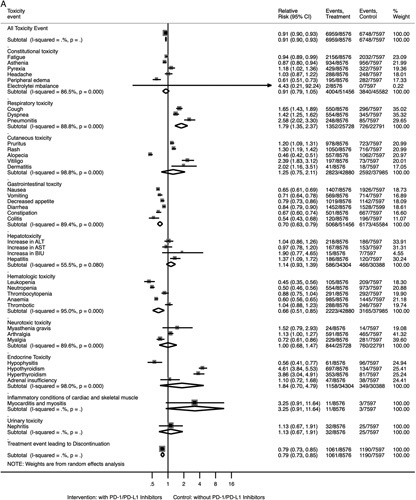

**Figure:**
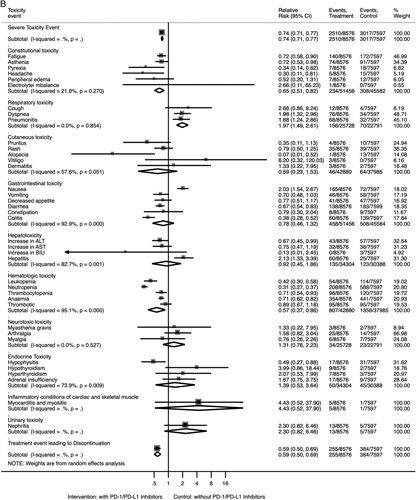

### Subgroup review by treatment regimens in the intervention arm

Compared with the control arm, patients treated with single PD-1/PD-L1 inhibitors (without chemotherapy) were less likely to experience overall toxicity (all grades: RR 0.86, 95% CI 0.80–0.92; grades 3–4: RR 0.56, 95% CI 0.42–0.75). System/organ-specifically, they were less likely to experience fatigue, asthenia, headache, peripheral edema, nausea, vomiting, decreased appetite, diarrhea, constipation, leukopenia, thrombocytopenia, anemia, thrombotic, and treatment event leading to discontinuation at any grade; and more likely for pneumonitis, pruritus, rash, vitiligo, hypothyroidism, and hyperthyroidism at any grade (all *P*<0.05; Supplementary Table S4, Supplemental Digital Content 3, http://links.lww.com/JS9/A415 and Supplementary Fig. S1A, Supplemental Digital Content 3, http://links.lww.com/JS9/A415). Compared with the control arm, patients treated with PD-1/PD-L1 inhibitors plus chemotherapy in the intervention arm were more likely to experience headache, pneumonitis, pruritus, rash, vomiting, diarrhea, colitis, thrombocytopenia, thrombotic, arthralgia, hypothyroidism, hyperthyroidism, hypophysitis, and treatment event leading to discontinuation at any grade (all *P*<0.05; Supplementary Table S4, Supplemental Digital Content 3, http://links.lww.com/JS9/A415 and Supplementary Fig. S1C, Supplemental Digital Content 3, http://links.lww.com/JS9/A415). The results of toxicities in grades 3–4 were shown in Supplementary Figure S1B, Supplemental Digital Content 3, http://links.lww.com/JS9/A415 and Supplementary Figure S1D, Supplemental Digital Content 3, http://links.lww.com/JS9/A415, respectively.

### Subgroup review by treatment regimens in the control arm

Compared with patients treated with only conventional chemotherapies (without CTLA-4 inhibitors) in control arms, patients treated with PD-1/PD-L1 inhibitors were at lower risks of having overall toxicities of any type (all grades: RR 0.87, 95% CI 0.81–0.93; grades 3–4: RR 0.63, 95% CI 0.52–0.75). Specifically, they were at lower risks for fatigue, asthenia, peripheral edema, nausea, vomiting, decreased appetite, constipation, leukopenia, neutropenia, thrombocytopenia, anemia, and myalgia at any grade; and were at higher risks for pneumonitis, pruritus, rash, vitiligo, colitis, increase in alanine aminotransferase (ALT), increase in aspartate aminotransferase (AST), hypothyroidism, hyperthyroidism, and hypophysitis at any grade (all *P*<0.05; Supplementary Table S4, Supplemental Digital Content 3, http://links.lww.com/JS9/A415 and Supplementary Fig. S2A, Supplemental Digital Content 3, http://links.lww.com/JS9/A415). In contrast, compared with patients treated by CTLA-4 inhibitors in control arms, patients in intervention arms were less likely for headache, pruritus, nausea, diarrhea, hypophysitis, and treatment event leading to discontinuation; and were more likely for fatigue, asthenia, vitiligo, arthralgia, hypothyroidism, and hyperthyroidism (all *P*<0.05; Supplementary Table S4, Supplemental Digital Content 3, http://links.lww.com/JS9/A415 and Supplementary Fig. S2C, Supplemental Digital Content 3, http://links.lww.com/JS9/A415). The results of toxicities at grades 3–4 for patients treated with only conventional chemotherapies in the control arm and patients treated with CTLA-4 inhibitors in the control arm were shown in Supplementary Figure S2B, Supplemental Digital Content 3, http://links.lww.com/JS9/A415 and Supplementary Figure S2D, Supplemental Digital Content 3, http://links.lww.com/JS9/A415, respectively.

### Subgroup review by PD-1/PD-L1 inhibitor types

Compared with patients in control arms, patients treated with nivolumab were less likely to experience overall toxicity (all grades: RR 0.88, 95% CI 0.80–0.95; grades 3–4: RR 0.57, 95% CI 0.39–0.81), gastrointestinal toxicity (including nausea, vomiting, decreased appetite, diarrhea, constipation, and colitis), hematologic toxicity (including neutropenia, thrombocytopenia, and anemia), hypophysitis, and treatment event leading to discontinuation at any grade; and were more likely for pruritus, vitiligo, hypothyroidism, and hyperthyroidism at any grade (all *P*<0.05; Supplementary Table S4, Supplemental Digital Content 3, http://links.lww.com/JS9/A415 and Supplementary Fig. S3A, B, Supplemental Digital Content 3, http://links.lww.com/JS9/A415). The overall comparison spectrum for pembrolizumab was similar to that for nivolumab, except for patients treated with pembrolizumab were more likely to develop pneumonitis, rash, and hepatitis (all *P*<0.05; Supplementary Table S4, Supplemental Digital Content 3, http://links.lww.com/JS9/A415 and Supplementary Fig. S3C, Supplemental Digital Content 3, http://links.lww.com/JS9/A415); and there was no significant difference between two arms for diarrhea, constipation, thrombocytopenia, hypophysitis, and treatment event leading to discontinuation (all *P*>0.05). The results of toxicities at grades 3–4 for nivolumab and pembrolizumab were shown in Supplementary Figure S3B, Supplemental Digital Content 3, http://links.lww.com/JS9/A415 and Supplementary Figure S3D, Supplemental Digital Content 3, http://links.lww.com/JS9/A415, respectively. The comparison results for PD-L1 inhibitors were shown in Supplementary Table S4, Supplemental Digital Content 3, http://links.lww.com/JS9/A415 and Supplementary Figure S3E, Supplemental Digital Content 3, http://links.lww.com/JS9/A415 and Supplementary Figure S3F, Supplemental Digital Content 3, http://links.lww.com/JS9/A415.

Previous research suggested that toxicity profiles of PD-1 and PD-L1 inhibitors were comparable, with overall toxicity incidences of 64 and 66%, respectively (*P*=0.8)^80,81^. However, possibly due to the benefit of having an inclusive and comprehensive research context (e.g. the most extensive list of toxicity types studied by far), findings of our review showed that although PD-1 inhibitors did not increase or decrease the incidence of headache and cough in cancer patients, incidences of these two toxicities increased significantly in patients who received a PD-L1 inhibitor-based treatment. Interestingly, the results of colitis were different among different PD-1/PD-L1 inhibitors. Patients receiving nivolumab were more likely to experience colitis when compared to the control arm (Supplementary Fig. S3A, Supplemental Digital Content 3, http://links.lww.com/JS9/A415); while patients receiving PD-L1 inhibitors were less likely to experience it (Supplementary Fig. S3E, Supplemental Digital Content 3, http://links.lww.com/JS9/A415). There was no difference in the colitis incidence between the intervention and control arm for patients receiving pembrolizumab (Supplementary Fig. S3C, Supplemental Digital Content 3, http://links.lww.com/JS9/A415). These findings expanded our understanding of PD-1/PD-L1 inhibitors-related toxicities in cancer patients and in turn, enriched the literature.

Results of our study indicated that nivolumab and pembrolizumab had noticeably different toxic effects on patients. A few possible explanations can shed light on this phenomenon. The first rationale is that while nivolumab and pembrolizumab share similarities (e.g. target epitopes on the PD-1 molecule and both belong to the IgG4 subclass), differences between them, such as varied antibody molecular properties, might have played a role in shaping their toxic effects. Considering that the epitope determines the drugs’ molecular target and, subsequently, its mode of action, it is probable that the epitope difference between these two drugs may influence their on-target side effects. Another possible explanation centers on the fact that the strength with which the antibody binds the target molecule has the potential to alter on-target side effects. As nivolumab and pembrolizumab’s affinities with recombinant human PD-1 protein (surface plasmon resonance) are different (nivolumab Kd=3.06 pM vs. pembrolizumab Kd=29 pmol/l), it is logical that these drugs could yield varied toxic effects. Also, as antibody specificity decreases, off-target side effects might become more likely. While there is a lack of data for pembrolizumab, available insights indicate that no binding to other members of the superfamily was found for nivolumab, including CD28 (cluster of differentiation 28), ICOS (inducible T cell co-stimulator), CTLA-4 (cytotoxic T-lymphocyte antigen 4), and BTLA (B- and T-lymphocyte attenuator) [ELISA (enzyme-linked immunosorbent assay)]^82^. This, in turn, suggests that differences in toxic effects between nivolumab and pembrolizumab might be a result of off-target side effects.

### Subgroup review by cancer histotype

From Supplementary Table S4, Supplemental Digital Content 3, http://links.lww.com/JS9/A415 and Supplementary Figure S4A–D, Supplemental Digital Content 3, http://links.lww.com/JS9/A415, 30.40% of melanoma patients and 42.82% of lung cancer patients treated with PD-1/PD-L1 inhibitors developed toxicities. For most toxicity types, the incidence proportions for lung cancer patients were higher than those for melanoma patients, while for some cutaneous toxicities (like pruritus, rash, and vitiligo), colitis, increase in BIU, and arthralgia, melanoma patients were higher (Supplementary Fig. S4, Supplemental Digital Content 3, http://links.lww.com/JS9/A415). For lung cancer, patients treated with PD-1/PD-L1 inhibitors were at lower risks of having toxicities of fatigue, asthenia, nausea, decreased appetite, leukopenia, neutropenia, and anemia; and were at higher risks of having pneumonitis, pruritus, rash, increase in ALT, increase in AST, hepatitis, hypothyroidism, hyperthyroidism, and hypophysitis at any grade (*P*<0.05; Supplementary Table S4, Supplemental Digital Content 3, http://links.lww.com/JS9/A415 and Supplementary Fig. S5A, B, Supplemental Digital Content 3, http://links.lww.com/JS9/A415). For melanoma, patients treated with PD-1/PD-L1 inhibitors were at lower risks than in control arms for having toxicities of headache, nausea, vomiting, decreased appetite, diarrhea, leukopenia, and thrombocytopenia; and were at higher risks of having dyspnea, vitiligo, dermatitis, hypothyroidism, and hyperthyroidism at any grade (all *P*<0.05; Supplementary Table S4, Supplemental Digital Content 3, http://links.lww.com/JS9/A415 and Supplementary Fig. S5C, Supplemental Digital Content 3, http://links.lww.com/JS9/A415). The results of toxicities at grades 3–4 for lung cancer and melanoma were shown in Supplementary Figure S5B, Supplemental Digital Content 3, http://links.lww.com/JS9/A415 and Supplementary Figure S5D, Supplemental Digital Content 3, http://links.lww.com/JS9/A415, respectively. As findings suggested above, although the overall toxicity trends were similar between melanoma and lung cancer, the respect incidences and specific toxicity types with significant differences were not exactly the same, probably due to the following three influence factors: first, patients with different cancers had different homeostasis points and tumor microenvironments, suggesting that their responses to drugs might be different; second, toxicity-related cellular/molecular targets and signal transduction pathways might be different in different cancers; third, the control arm at lung cancer RCTs usually involved cytotoxic chemotherapies, whereas, for melanoma, ipilimumab was the more common choice. Because of the small amount of literature, we did not analyze some cancer types, such as gastrointestinal cancer, genitourinary cancer, and HNSCC, separately.

According to the results, patients treated with PD-1/PD-L1 inhibitors were at lower risks of having toxicities of fatigue, asthenia, peripheral edema, nausea, vomiting, decreased appetite, diarrhea, constipation, leukopenia, neutropenia, anemia, arthralgia, myalgia, and treatment event leading to discontinuation; and were at higher risks to have pneumonitis, pruritus, hypothyroidism, hyperthyroidism at any grade (all *P*<0.05; Supplementary Table S4, Supplemental Digital Content 3, http://links.lww.com/JS9/A415 and Supplementary Fig. S5E, F, Supplemental Digital Content 3, http://links.lww.com/JS9/A415). However, there was little research on some other cancers, such as sarcoma. We reviewed relative literature. One study indicated that PD-L1 mRNAs were highly expressed in osteosarcoma tissues; the combination of anti-PD-1 antibody and cisplatin significantly decreased the proliferation and increased the apoptosis of K7M2 cells in a coculture system^83^.

## Some other potential immune targets and relative preclinical research

Tumors evade immune-mediated recognition by a variety of immune escape mechanisms. During chronic tumor antigen exposure, T cell dysfunction or failure occurs, and various checkpoint inhibitory receptors (IRs) limit T cell survival and function. The immune checkpoint inhibitors (PD-1/PD-L1 inhibitors) hold great promise for cancer treatment and have provided ample evidence of clinical benefits in many solid tumors. Apart from these immune checkpoint blockades, several other IRs were blocked in clinical practice. Firstly, the T cell immunoreceptor with immunoglobulin and immunoreceptor tyrosine-based inhibitory motif (ITIM) domain (TIGIT) is a promising new target for cancer immunotherapy^84^. TIGIT was expressed by tumor cells and antigen-presenting cells in the tumor microenvironment; and was upregulated by immune cells, including natural killer cells, regulatory T cells, and activated T cells^85^. Multiple lines of evidence support that TIGIT plays an important role in innate immunity and is adaptive against tumors, such as pancreatic cancer, melanoma, and bladder cancer^86–88^. Interestingly, dual PD-L1/TIGIT blockade (atezolizumab/tiragolumab) appears to provide superior clinical benefits as compared with PD-L1 blockade alone as a first-line therapy for patients with PD-L1-positive non-small cell lung cancers, despite similar toxicity profiles^89^. Secondly, proprotein convertase subtilisin/kexin type 9 (PCSK9), which is approved for hyperlipidemia treatment, has the potential to boost the anticancer efficacy of ICIs^90^. The results also indicated that PCSK9, as a key protein in regulating cholesterol metabolism, can boost tumor response to immune checkpoint therapy and also enhance the efficacy of anti-PD-1 immune checkpoint therapy significantly^91^. Moreover, the results indicated that combined anti-PD-1 antibodies with either evolocumab or alirocumab had demonstrated potent tumor-suppressing efficacy, and PCSK9 inhibitors may enhance immune checkpoint blockade therapy without additional side effects^91^. Thirdly, mesothelin is a tumor antigen which is highly expressed in malignant tumors, such as lung adenocarcinoma, pancreatic cancer, ovarian cancer, and mesothelioma^92^. It is an attractive target for cancer immunotherapy because its normal expression is limited to mesothelial cells, which are dispensable^93^. Furthermore, the phase II randomized clinical trials of CRS-207 were as an enhancer and combined with an immune checkpoint to inhibit pancreatic cancer^93^. Fourthly, melanocortin 5 receptor (MC5R), which is a G protein-coupled receptor (GPCR), is expressed in the terminally differentiated sebaceous gland^94^. One study indicated that MC5R is a potential target for cancer immunotherapy, and MC5R peptide antagonists boosted antitumor immunity and anti-programmed cell death protein 1 (anti-PD-1) immunotherapy^95^. Finally, discoidin domain receptor 1 (DDR1), a collagen receptor with tyrosine kinase activity, instigates immune exclusion by promoting collagen fiber alignment^96^. The DDR1 extracellular domain (DDR1-ECD) is required for immune exclusion, and DDR1-ECD is sufficient to rescue the growth of DDR1 knockout tumors in immunocompetent hosts^97^. Hence, the development of PD-1/PD-L1 target drugs promotes the discovery of more new tumor immune targets. The new tumor immune targets discovered include immune checkpoint molecules and epigenetic and immunological targets. The discovery and development of these new tumor immune targets will provide more new programs for tumor immunotherapy.

## Strengths and limitations

To establish a clinically relevant landscape of PD-1/PD-L1 inhibitors-related side effects, we included a total of 11 categories and 39 types of toxicities, the most extensive list studied by far, to examine the interplay between key cancer treatment considerations (i.e. cancer type, choice of drug, exposure to chemotherapy, and therapy regimen) and toxicities associated with PD-1/PD-L1 inhibitors. In other words, one key crown jewel of our review was that we not only examined the overall toxicities but also thoroughly capitalized on heterogeneities across included studies – we investigated these differences and provided critical findings on how investigations on heterogeneities in subgroups can inform research and practice. Specifically, we conducted subgroup reviews of toxicities associated with PD-1/PD-L1 inhibitors from the perspectives of affected systems/organs, toxicity types, toxicity grades, cancer types, drug choices, exposure to chemotherapy, and therapy regimens – analyses that were absent from the current literature. Based on insights gained from the subgroup reviews, we were able to provide novel and comprehensive insights on the toxicities of PD-1/PD-L1 inhibitors to the literature. The funnel plot for all included 29 studies included in the meta-analysis can be seen in Supplementary Figure S6. While addressing substantial gaps in the literature, this systematic review was not without limitations. First, for starters, as the present research is a meta-analysis at the study level rather than at the patient level, insights on risk factors associated with the development of toxicities cannot be found in our study. Second, there was a possible overlap in CTCAE definitions which prevents understanding the true rates of specific toxicities. Third, we pooled data from studies that used different PD-1/PD-L1 inhibitors at variable doses, so we may have missed differences in toxicity rates across drugs or based on dosage differences. Given the wide variation in drug and dose across studies, we were unable to perform subgroup analyses to examine these factors. However, we found little heterogeneity across studies for toxicity outcomes, suggesting little difference based on the specific drug or dose. Also, anti-PD-1/PD-L1 toxicity and efficacy were considered not dose-dependent once above a level of 1 mg/kg. Fourth, although we analyzed several different cancer types, especially melanoma and lung cancer, there were still many other cancer types not enrolled (the search strategy could be referred to Supplementary Table S7, Supplemental Digital Content 3, http://links.lww.com/JS9/A415). In further studies, we will update the meta-analysis with the increase of relevant RCT studies. Fifth, owing to the short interval of follow-up data currently available from clinical trials and a lack of clarity in the systematic capture of many toxicities, we are likely to have underestimated the true rates of toxicities.

## Conclusion and perspectives

Based on the largest and most comprehensive series of subgroup analyses, our meta-analysis has shed light on differences in cancer patients’ risk of experiencing PD-1/PD-L1-related toxicities. These analyses offered novel insights on the incidence differences of PD-1/PD-L1-related toxicities that are associated with the affected systems/organs, toxicity types, toxicity grades, cancer types, drug choices, exposure to chemotherapy, and therapy regimens. Overall, our study can help establish a clinically relevant landscape of side effects associated with anti-PD-1/PD-L1 agents and in turn, help inform clinicians and oncologists in their research and practice in the field of cancer research and beyond. For instance, when we have known relatively accurately that a certain PD-1/PD-L1 inhibitor is more likely to cause certain toxic side effects of a certain system or a certain organ when treating certain types of cancer, clinicians could carry out relevant clinical tests in a more targeted and more timely manner or give patients specific preventive measures in advance, so that the possible side effects might be minimized, and to a certain extent, patients might be relieved, and medical resources might be saved.

To further extend the literature, future research could focus on using effective targeted measures to decrease levels of immunology-related toxicities cancer patients face and in turn, improve treatment efficacy as well as patients’ well-being and quality of life.

## Ethical approval

The analysis followed the Strengthening the Reporting of Observational Studies in Epidemiology (STROBE) guidelines. National Cancer Center/National Clinical Research Center for Cancer/Cancer Hospital, Chinese Academy of Medical Sciences, and Peking Union Medical College institutional review board approved this study as exempt.

## Sources of funding

This work was supported by the China National Key R&D (or Research and Development) Program (No. 2020AAA0105000 and 2020AAA0105004), the Natural Science Foundation of China (No. 82173328), the Natural Science Foundation of China (No. 81872160), the Natural Science Foundation of China (No. 82072940), the Natural Science Foundation of China (No. 82103047), the Natural Science Foundation of China (No. 82102887), the CAMS Innovation Fund for Medical Sciences (CIFMS) (No. 2021-I2M-C&T-A-012), the CAMS Innovation Fund for Medical Sciences (CIFMS) (No. 2021-I2M-C&T-B-044), the CAMS Innovation Fund for Medical Sciences (CIFMS) (No. 2022-I2M-C&T-B-087), the Non-profit Central Research Institute Fund of Chinese Academy of Medical Sciences (No. 2022-JKCS-04), the Beijing Association for Science and Technology's “Golden-Bridge Seed Funding Program” (No. ZZ22027), the Beijing Hope Run Special Fund of Cancer Foundation of China (No. LC2020L01), the Beijing Hope Run Special Fund of Cancer Foundation of China (No. LC2019L07), the Beijing Hope Run Special Fund of Cancer Foundation of China (No. LC2022A19), the Chinese Young Breast Experts Research project (No. CYBER-2021-005), the XianSheng Clinical Research Special Fund of China International Medical Foundation (No. Z-2014-06-2103), the Beijing Xisike Clinical Oncology Research Foundation (No. Y-Young2021-0017), and the Beijing Xisike Clinical Oncology Research Foundation (No. Y-NESTLE2022QN-0018).

## Author contribution

X.K., Y.F., J.G., and J.W.: conceptualization; X.K. and L.C.: data collection; L.Z., Y.H., Z.Q., and Y.L.: methodology; L.Z. and R.J.S.: supervision; X.K. and L.Z.: writing – original draft; Z.S., R.J.S., X.K., and L.Z.: writing – review and editing. All authors have read and agreed to the published version of the manuscript.

## Conflicts of interest disclosure

The authors declare no conflicts of interest.

## Research registration unique identifying number (UIN)

1. Name of the registry: PROSPERO.
2. Unique identifying number or registration ID: CRD42019135113.
3. Hyperlink to your specific registration (must be publicly accessible and will be checked): https://www.crd.york.ac.uk/prospero/display_record.php?RecordID=135113

## Provenance and peer review

Not commissioned, externally peer-reviewed.

## Supplementary Material
